# Interleukin-27R Signaling Mediates Early Viral Containment and Impacts Innate and Adaptive Immunity after Chronic Lymphocytic Choriomeningitis Virus Infection

**DOI:** 10.1128/JVI.02196-17

**Published:** 2018-05-29

**Authors:** James A. Harker, Kurt A. Wong, Simone Dallari, Phuc Bao, Aleksandr Dolgoter, Yeara Jo, Ellen J. Wehrens, Monica Macal, Elina I. Zuniga

**Affiliations:** a Molecular Biology Section, Division of Biological Sciences, University of California San Diego, La Jolla, California, USA; b Section of Inflammation, Repair and Development, National Heart and Lung Institute, Imperial College London, London, United Kingdom; Icahn School of Medicine at Mount Sinai

**Keywords:** chronic infection, DCs, IL-27, LCMV, T cells, antibody function, cytokines, dendritic cells, type I IFN

## Abstract

Chronic viral infections represent a major challenge to the host immune response, and a unique network of immunological elements, including cytokines, are required for their containment. By using a model persistent infection with the natural murine pathogen lymphocytic choriomeningitis virus clone 13 (LCMV Cl13) we investigated the role of one such cytokine, interleukin-27 (IL-27), in the control of chronic infection. We found that IL-27 receptor (IL-27R) signaling promoted control of LCMV Cl13 as early as days 1 and 5 after infection and that *il27p28* transcripts were rapidly elevated in multiple subsets of dendritic cells (DCs) and myeloid cells. In particular, plasmacytoid DCs (pDCs), the most potent type 1 interferon (IFN-I)-producing cells, significantly increased *il27p28* in a Toll-like receptor 7 (TLR7)-dependent fashion. Notably, mice deficient in an IL-27-specific receptor, WSX-1, exhibited a pleiotropy of innate and adaptive immune alterations after chronic lymphocytic choriomeningitis virus (LCMV) infection, including compromised NK cell cytotoxicity and antibody responses. While, the majority of these immune alterations appeared to be cell extrinsic, cell-intrinsic IL-27R was necessary to maintain early pDC numbers, which, alongside lower IFN-I transcription in CD11b^+^ DCs and myeloid cells, may explain the compromised IFN-I elevation that we observed early after LCMV Cl13 infection in IL-27R-deficient mice. Together, these data highlight the critical role of IL-27 in enabling optimal antiviral immunity early and late after infection with a systemic persistent virus and suggest that a previously unrecognized positive-feedback loop mediated by IL-27 in pDCs might be involved in this process.

**IMPORTANCE** Persistently replicating pathogens, such as human immunodeficiency virus, hepatitis B virus, and hepatitis C virus, represent major health problems worldwide. These infections impose a long-term challenge on the host immune system, which must be heavily and continuously regulated to keep pathogen replication in check without causing fatal immunopathology. Using a persistently replicating rodent pathogen, LCMV, in its natural host, we identified the cellular sources and effects of one important regulatory pathway, interleukin-27 receptor WSX-1 signaling, that is required for both very early and late restriction of chronic (but not acute) infection. We found that WSX-1 was necessary to promote innate immunity and the development of aberrant adaptive immune responses. This not only highlights the role of IL-27 receptor signaling in regulating distinct host responses that are known to be necessary to control chronic infections, but also positions IL-27 as a potential therapeutic target for their modulation.

## INTRODUCTION

Chronic infections with persistently replicating viruses, which include the major human pathogens human immunodeficiency virus type 1 (HIV-1), hepatitis C virus (HCV), and hepatitis B virus (HBV), present the host with a lifelong challenge. As a consequence, infection by these pathogens often results in heavily regulated immune responses that aim to keep the persistent virus in check without causing fatal immunopathology. Such immune regulation during chronic viral infections includes hypofunctionality of CD4^+^ and CD8^+^ T cells and delayed antibody production, as well as rapidly attenuated, but sustained, type I IFN (IFN-I) responses (reviewed in reference 1).

Lymphocytic choriomeningitis virus (LCMV) is a small, segmented RNA virus of the family Arenaviridae that cause natural, vertically transmitted, persistent infections in selected rodent hosts. LCMV has a strain-dependent capacity to cause either acute, e.g., LCMV Armstrong 53b (ARM), or chronic, e.g., LCMV clone 13 (Cl13), systemic infection in adult mice (2). Chronic infection of mice with LCMV Cl13 results in a systemic infectiont sharing many common immunological features with persistent human infections, which is eventually cleared from the majority of tissues by 100 days postinfection (p.i.) (1). Clearance of LCMV Cl13 requires a combined effort of innate B and T cell-mediated immunity, as defects in any of the arms of the immune system result in lifelong viremia (3–5).

Cytokine signaling can play pivotal roles in both promoting viral persistence and eventual control of LCMV. Increased signaling via interleukin-10 (IL-10) and transforming growth factor beta (TGF-β) has been described during chronic LCMV infection and can dampen T cell responses (6–9). Exhausted virus-specific T cells also become less responsive to the critical γc survival cytokines IL-2, IL-7, and IL-15 (10–12), although exogenous IL-2 and IL-7 can be used therapeutically to promote virus control in an established LCMV Cl13 infection (10, 13). IL-21, another γc cytokine, is crucial for maintenance of virus-specific CD8^+^ T cell numbers during LCMV Cl13 infection (14–16). Meanwhile, IL-6 is critical for maintaining virus-specific CD4^+^ T cell responses by promoting T follicular helper cell (T_FH_) differentiation and virus-specific antibody (17). The type I interferons IFN-α and -β are rapidly elevated and subsequently attenuated after chronic LCMV infection, playing an important, though complex, role in direct viral control and orchestration of immune responses (18–23).

IL-27 is a heterodimeric cytokine comprised of IL-27p28 and EBI3 subunits, making it structurally related to the IL-12 family of cytokines (reviewed in reference 24). It signals through the common IL-6 cytokine family signal transduction molecule gp130 in conjunction with a cytokine-specific receptor, WSX-1 (encoded by *Il27ra*). In accordance with IL-27's use of gp130, downstream signaling primarily occurs through phosphorylation of STAT-1 and STAT-3, but IL-27 can also cause the phosphorylation of STAT-2, -4, and -5 (25, 26). WSX-1 is highly expressed on T cells, and initial studies on the biological role of IL-27 focused on CD4^+^ and CD8^+^ T cells (27). *In vitro*, IL-27 can upregulate CD8^+^ T cell expression of IFN-γ and granzyme B, enhancing cytotoxic function (28), while on CD4^+^ T cells, it promotes increased Tbet expression and T helper 1 (Th1) differentiation (26). In regulatory T cells (Tregs), IL-27 can regulate distinct functional characteristics (29, 30), while in helper T cells it is also associated with the production of IL-21 and suppression of Th17 responses (31–34). During peptide vaccination, IL-27 can be important in the generation of T_FH_s (34). In contrast, IL-27 signaling can also promote IL-10 production by CD4 T cells *in vivo* (35, 36), in part via upregulation of Blimp-1, a transcriptional antagonist of T_FH_ differentiation (37). IL-27 also influences other immune cells, regulating natural killer (NK) cell cytotoxicity and cytokine secretion (38); upregulating CD39 on conventional dendritic cells (DCs), which results in enhanced suppression of T cell responses (39); and inhibiting viral replication in HIV- and HCV-infected cells *in vitro* (40–42).

In contrast to their wild-type (WT) counterparts, WSX-1-deficient mice develop lifelong viremia after LCMV Cl13 infection (43). While intrinsic WSX-1 signaling is required for the optimal accumulation and maintenance of virus-specific CD4^+^ T cells, CD4 T cell-extrinsic mechanisms cause enhanced numbers of virus-specific CD4^+^ T cells in WSX-1-deficient mice infected with LCMV Cl13, suggesting additional mechanisms underlying the lack of virus control in nonchimeric mice (43). In this study, we found that IL-27 expression was rapidly increased after LCMV Cl13 infection. Specifically, IL-27 was elevated in conventional DCs (cDCs), plasmacytoid DCs (pDCs), and macrophages, and this was fully dependent on Toll-like receptor 7 (TLR7) in pDCs but TLR7 independent in cDCs and macrophages. Loss of IL-27 signaling resulted in reduced IFN-I and dysregulated DC and NK cell numbers and/or activation, which correlated with a diminished capacity to control LCMV Cl13 early after infection. Although ubiquitous WSX-1 deficiency led to enhanced virus-specific CD8^+^ T cell and plasmablast numbers, virus-specific antibody responses were diminished at later stages of infection, providing a possible explanation for the failure to control persistent replication late after infection. Importantly, mixed chimeras indicated that, while most of the above-mentioned immune alterations were cell extrinsic, IL-27 directly contributed to pDC accumulation early after chronic LCMV infection. These results reveal a previously unappreciated role for IL-27 in orchestrating early innate antiviral responses and late humoral immunity, which are associated with very early and late containment of a systemic persistent infection. In addition, our results suggest a novel DC positive-feedback loop, in which viral infection rapidly promotes production of DC- and macrophage-derived IL-27, directly promoting pDC accumulation and enhancing IFN-I transcription in cDCs early after infection.

## RESULTS

### IL-27 promotes early control of LCMV clone 13 infection.

**FIG 1:**
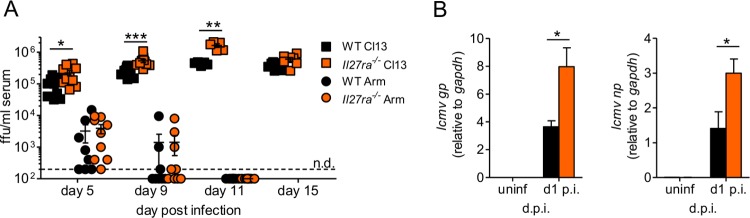
*Il27ra*^−/−^ mice had increased viral loads early after chronic, but not acute, LCMV infection. WT and *Il27ra*^−/−^ mice were infected with 2 × 10^6^ PFU of LCMV Cl13 i.v. (A) Serum viral loads were determined at multiple times postinfection and compared with those of WT and *Il27ra*^−/−^ mice infected with 2 × 10^6^ PFU LCMV Arm i.v. ffu, focus-forming units. The data represent the results of 2 independent experiments; *n* = 10 mice per time point per group. (B) *lcmv gp* and *np* expression levels were determined in the spleen homogenates of LCMV Cl13-infected WT and *Il27ra*^−/−^ mice 24 h postinfection. The data are representative of the results of 2 independent experiments; *n* = 5 mice per group; d.p.i., days p.i. The error bars indicate SEM. n.d., not determined. ***, *P* < 0.001; **, *P* < 0.01; *, *P* < 0.05.

We have previously reported that WSX-1 is not only required for long-term control of LCMV Cl13, but also seems to regulate the viral burden earlier in infection (43). In confirmation of this, we found that *Il27ra*^−/−^ mice had significantly increased viremia at days 5, 9, and 11 p.i. with LCMV Cl13 compared to WT mice, with similar viral loads seen at day 15 (Fig. 1A). Importantly, in contrast, there was no apparent defect in the ability of *Il27ra*^−/−^ mice to control LCMV ARM, with viremia being undetectable by day 11 p.i. Viral loads at time points earlier than day 5 p.i. are difficult to determine, especially within the first 24 h postinfection. The expression of both *lcmv gp* and *np* in spleen homogenates, however, was elevated in the absence of WSX-1 24 h after LCMV Cl13 infection (Fig. 1B).

Taken together, these data showed that IL-27 signaling was critical in promoting persistent virus control from a very early time point p.i. and appeared essential for long-term control of persistent (but not acute) LCMV infection (Fig. 1A) (43).

### Systemic viral infection rapidly induces IL-27 upregulation.

Viral infection often results in the rapid production and release of many cytokines and chemokines, which prime and instruct the immune system. Many of these mediators are quickly dampened, while others are produced at heightened concentrations throughout infection or undergo several phases of production (17). Given that IL-27 signaling is crucial to contain the magnitude of early LCMV Cl13 replication (Fig. 1A and B), we sought to determine the timing and cellular source of IL-27 production early during LCMV Cl13 infection.

**FIG 2:**
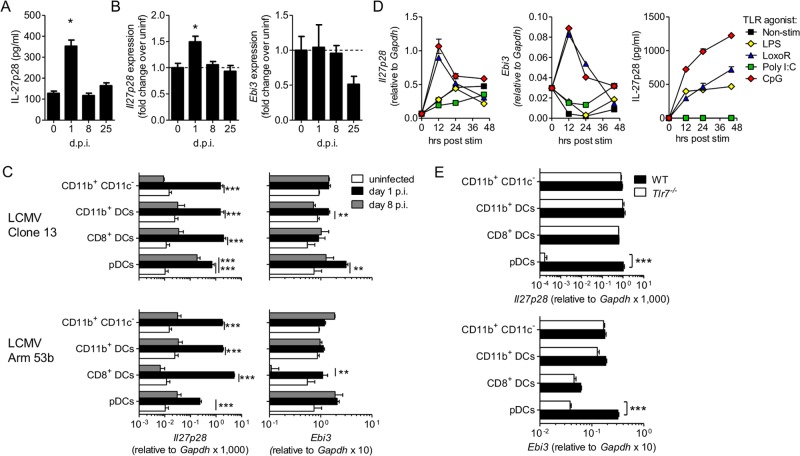
Dendritic cells and other myeloid cells were major producers of IL-27 within 24 h after LCMV infection *in vivo*. (A and B) Wild-type C57B/6 mice were infected with 2 × 10^6^ PFU of LCMV Cl13 i.v., and the concentration of IL-27p28 (A) and expression of *Il27p28* and *Ebi3* (B) were determined in spleen homogenates at days 1, 8, and 25 p.i. and compared to the levels in uninfected (unif) mice. (C) WT mice were infected with LCMV Cl13 or Arm 53b, and expression of *Il27p28* and *Ebi3* relative to *Gapdh* was determined in FACS-purified splenic pDCs (CD19^−^ Thy1.2^−^ NK1.1^−^ CD11c^+^ CD11b^−^ B220^+^ PDCA1^+^), CD8^+^ DCs (CD19^−^ Thy1.2^−^ NK1.1^−^ CD11b^−^ B220^−^ CD8^+^ CD11c^+^), CD11b^+^ DCs (CD19^−^ Thy1.2^−^ NK1.1^−^ CD8^−^ CD11b^+^ CD11c^+^), and myeloid cells (CD19^−^ Thy1.2^−^ NK1.1^−^ CD11c^−^ CD11b^+^). (D) *Il27p28* and *Ebi3* expression and IL27p28 protein levels were determined by quantitative PCR (qPCR) and by ELISA, respectively, in TLR agonist-stimulated DCs from BM-Flt3L cultures. (E) Expression of *Il27p28* and *Ebi3* determined as for panel C with WT or *Tlr7*^−/−^ mice at day 1 after LCMV Cl13 infection. The data are representative of the results of 3 (A and B) or 2 (D and E) independent experiments with ≥3 mice per group or cumulative for 4 independent repeats with ≥5 mice (C) The error bars indicate SEM. ***, *P* < 0.001; **, *P* < 0.01; *, *P* < 0.05.

IL-27 was undetectable in the serum at any time point analyzed (between day 1 and day 30 postinfection) after LCMV Cl13 infection (data not shown). Uninfected WT C57B/6 mice, however, did have detectable levels of IL-27 in whole spleens, which were increased at day 1 p.i. and returned to basal naive concentrations at days 8 and 25 p.i. (Fig. 2A). Expression of *Il27p28* transcript in the whole spleen mirrored the protein, with detectable transcripts in naive mice and significant increase at day 1, but not days 8 and 25, p.i. In contrast, splenic *Ebi3* expression was unchanged throughout infection or slightly reduced at day 25 p.i. (Fig. 2B).

**FIG 3:**
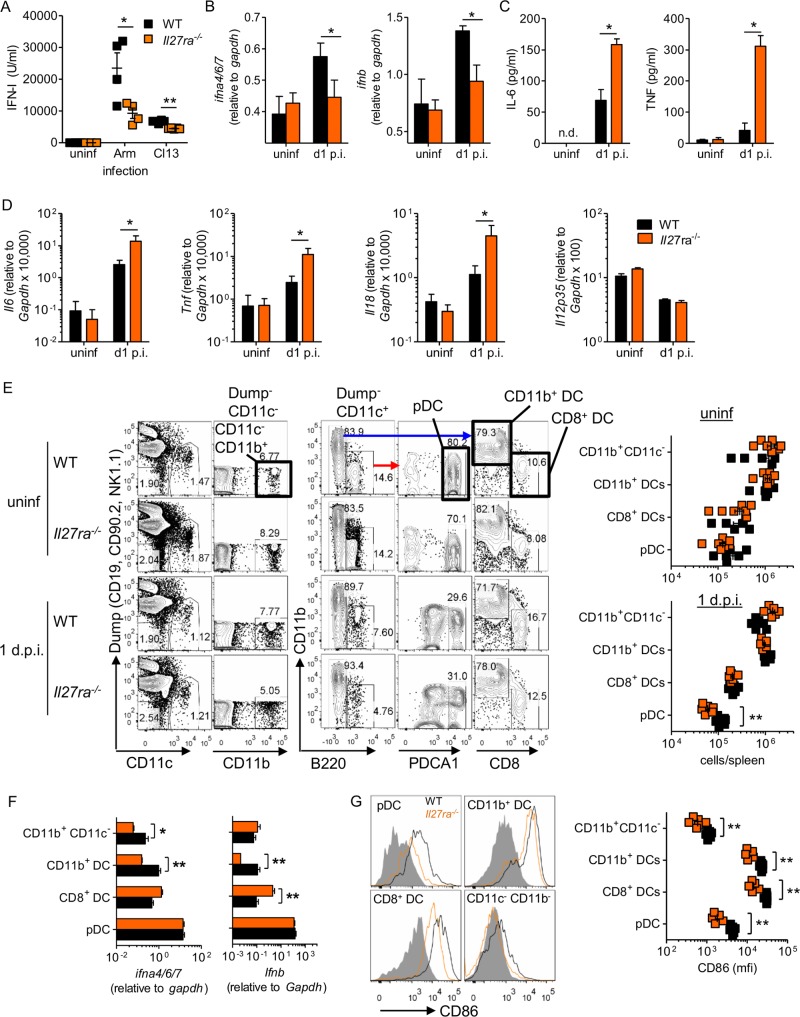
IL-27 regulates IFN-I and DC responses after LCMV infection. WT or *Il27ra*^−/−^ mice were infected with 2 × 10^6^ PFU of LCMV ARM or Cl13 (A) or left uninfected (A to G) and analyzed 24 h p.i. (A) Serum IFN-I measured by bioassay. (B) *Ifna4*, -*6*, and *-7* and *Ifnb* expression measured in spleen homogenates. (C) Serum IL-6 and TNF measured by ELISA. (D) *Il6*, *Tnf*, *Il18*, and *Il12p35* expression in spleen homogenates. (E) Number of splenic DCs determined by FACS. (F) *Ifna* and *Ifnb* expression determined in FACS-isolated DCs. (G) CD86 expression in DC subsets determined by flow cytometry. The data represent the results of 3 experiments with ≥4 mice per group. The error bars indicate SEM. **, *P* < 0.01; *, *P* < 0.05.

Having determined that IL-27 was elevated rapidly upon infection, we next investigated which cells might be responsible for its production. CD8^+^ DCs and macrophages have previously been reported to produce significant amounts of IL-27 (44, 45). As DCs and macrophages are highly activated in response to LCMV (22), we analyzed IL-27 production by these cells after LCMV infection, gating CD8^+^ DCs, CD11b^+^ DCs, pDCs, and CD11c^−^ CD11b^+^ cells (here referred to as myeloid cells; all the cell populations were gated as shown in Fig. 3E). There was detectable *Il27p28* expression in DC and myeloid subsets prior to infection, which was highly upregulated in the same cells 24 h p.i. (Fig. 2C). Across the 4 independent experiments analyzed, however, it was unclear if any single cell type upregulated *Il27p28* significantly above the other cell types, suggesting upregulation was similar in all the cell types. In contrast *Ebi3* expression was not dramatically upregulated after infection. Similar patterns of *Il27p28* elevation were seen in mice infected with the acute LCMV ARM at day 1 p.i. Interestingly, however, by day 8 postinfection, *Il27p28* expression was still increased in pDCs isolated from Cl13-infected, but not ARM-infected, mice. None of the other cell types examined showed increased *Il27p28* expression at the later time point in either infection.

Given that DCs rapidly produce IL-27 in response to LCMV infection *in vivo*, we next evaluated whether this could be TLR mediated. Flt3L differentiated bone marrow (BM) DCs, a mixture of pDCs and CD11b^+^ DCs, showed rapid upregulation of *Il27p28* and *Ebi3* in response to TLR7 (loxoribine) or TLR9 (CpG) agonists and to a lesser extent or not at all upon TLR4 (lipopolysaccharide [LPS]) stimulation (Fig. 2D). They did not respond to poly(I·C), a TLR3 agonist. Loxoribine, CpG, and LPS, but not poly(I·C), stimulation also led to detectable IL-27 protein in the culture supernatants (Fig. 2D). As pDCs had not previously been reported to produce IL-27 and had prolonged IL-27 expression after LCMV Cl13 infection, we evaluated whether TLR7 signaling, known to be essential for pDC IFN-I production during LCMV infection (22), played a role in the *in vivo* production of IL-27. Indeed, *Il27p28* and *Ebi3* upregulation was completely ablated in pDCs taken from *Tlr7*^−/−^ LCMV-infected animals (Fig. 2E). TLR7 deficiency did not, however, affect IL-27 expression by the other DC or myeloid cell types analyzed.

Taken together, these data indicated that IL-27 was produced by various innate cellular sources early after LCMV infection, and this elevation was mostly transient in DCs and macrophages but more sustained in pDCs after persistent LCMV infection. In particular, pDC (but not cDC) production of IL-27 was fully dependent on TLR7.

### IL-27 regulates early innate immune responses after LCMV infection.

LCMV infection results in rapid activation of the host's immune response, including production of high levels of IFN-I (22). Early IFN-I signaling can be vital in establishing an antiviral environment early during infection by the induction of gene networks known to perturb viral replication and to activate a range of immune cells (46). Compared to WT mice, *Il27ra*^−/−^ mice had significantly lower circulating bioactive IFN-I 24 h after infection with LCMV ARM or Cl13 (Fig. 3A). Absence of WSX-1 also resulted in failure to upregulate both *Ifna* and *Ifnb* in the spleen after LCMV Cl13 infection (Fig. 3B). In contrast, the proinflammatory mediators IL-6 and tumor necrosis factor (TNF) were both significantly increased in sera from *Il27ra*^−/−^ versus wild-type mice 24 h after Cl13 infection (Fig. 3C). *Il6*, *Tnf*, and *Il18* expression was also significantly higher in the spleens of *Il27ra*^−/−^ mice than in those of WT controls, but expression levels of *Il12p35* were similar in WT and WSX-1-deficient mice (Fig. 3D).

In LCMV infection, IFN-I is produced by both pDCs, via a TLR7-dependent pathway, and cDCs and macrophages, where production is dependent on MAVS and MDA5 (22, 47, 48). Uninfected *Il27ra*^−/−^ mice had numbers of pDCs, cDCs, and other myeloid cells similar to those of WT mice in the spleen (Fig. 3E). The numbers of CD8^+^ DCs, CD11b^+^ DCs, and CD11c^−^ CD11b^+^ myeloid cells in the spleen 24 h after infection were unaffected by *Il27ra* deletion; however, the number of pDCs at this time was significantly reduced, by approximately 2-fold, in *Il27*ra^−/−^ mice compared to WT mice (Fig. 3E). Expression of IFN-I genes by the different cell populations was variably affected by the absence of WSX-1. Other myeloid cells had significantly reduced *Ifna*, and CD11b^+^ DCs had significantly reduced *Ifna* and *Ifnb* expression, but CD8^+^ DCs had increased *Ifnb* expression (Fig. 3F). Meanwhile, *Ifna* and *Ifnb* expression by pDCs, which had the highest expression of both IFN-I genes on a per cell basis (22), was unaffected by WSX-1 deletion (Fig. 3F). In addition, upregulation of the costimulatory molecule CD86 was adversely affected in all four innate cell populations (Fig. 3G).

**FIG 4:**
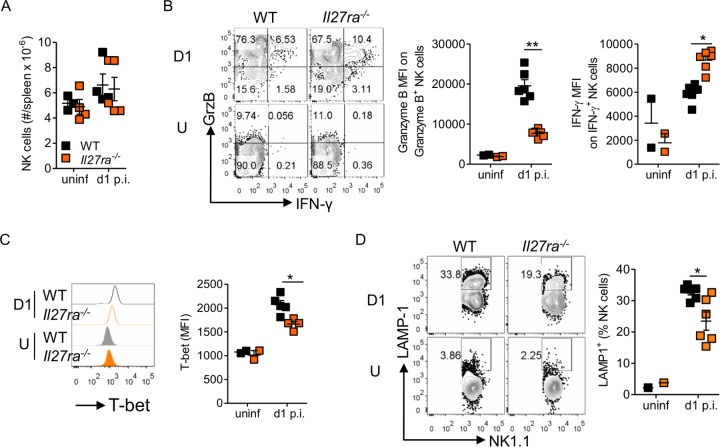
IL-27R signaling regulates NK cell function after LCMV infection. WT and *Il27ra*^−/−^ mice were infected with 2 × 10^6^ PFU of LCMV Cl13 i.v., analyzed at 24 h p.i. (D1), and compared to uninfected mice (U). Spleen NK cells (NK1.1^+^ CD3^−^ CD19^−^) were enumerated by flow cytometry (A), along with their expression of granzyme B (GrzB) and IFN-γ (B), Tbet (C), and LAMP-1 (D). Granzyme B, IFN-γ, and LAMP-1 were measured after incubation of splenocytes in the presence of brefeldin A for 5 h. The data are representative of 3 experiments with ≥4 mice per group. The error bars indicate SEM. **, *P* < 0.01; *, *P* < 0.05.

NK cells, responding to changes in the cytokine milieu, also promptly respond to LCMV infection. The numbers of NK cells in the spleen both prior to and 24 h after infection were unaffected by the deletion of *Il27ra* (Fig. 4A). Interestingly, however, the functionality of NK cells 24 h after LCMV Cl13 infection was altered, with *Il27ra*-^−/−^ NK cells showing reduced granzyme B and increased IFN-γ production compared to WT NK cells (Fig. 4B). Specifically, it appeared from the gating that the proportion of granzyme B-positive *Il27ra*^−/−^ NK cells was similar to that seen in WT NK cells (data not shown), but the per cell expression of granzyme B in granzyme B-positive NK cells was significantly lower (Fig. 4B). Likewise, while there was a small increase in the proportion of IFN-γ^+^ NK cells in *Il27ra*^−/−^ mice compared to WT mice, the biggest change was the overall amount of IFN-γ per NK cell (Fig. 4B). Surprisingly, given the increased IFN-γ production, NK cells from *Il27ra*^−/−^ mice had decreased Tbet, a known positive regulator of IFN-γ production, compared to WT mice (Fig. 4C). This was accompanied by reduced degranulation, as measured by LAMP-1 (Fig. 4D).

These data showed that signaling through IL-27 regulated a number of critical innate immune parameters very early after systemic LCMV infection. These changes should, however, be considered in the light of the increased viral RNA (Fig. 1B), suggesting more viral replication, which could impact the degree of stimulation or inhibition of a particular pathway in cells from *Il27ra*^−/−^ virus- versus WT-virus-infected mice.

### Direct IL-27 signaling favors pDC accumulation early after LCMV Cl13 infection.

**FIG 5:**
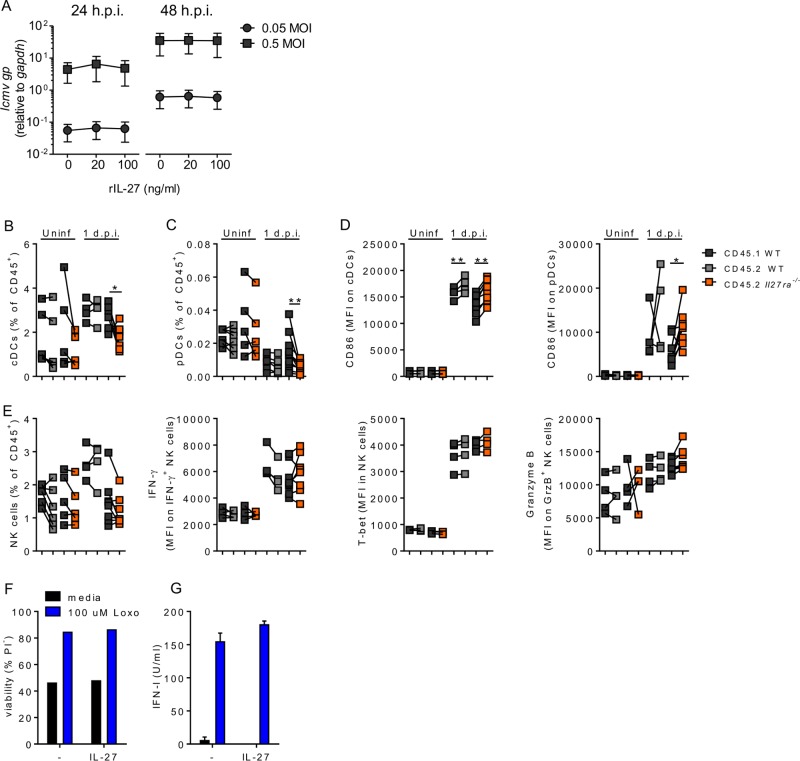
IL-27 has no direct antiviral effect, and extrinsic *Il27ra* signaling promotes optimal NK cell and DC activation after LCMV Cl13 infection. (A) A549 cells were infected at an MOI of 0.05 or 0.5 with LCMV Cl13 with or without treatment with increasing doses rIL-27 from 1 h prior to infection. *Lcmv gp* expression was determined by RT-qPCR. (B to D) WT:C57BL/6 or WT:*Il27ra*^−/−^ mixed bone marrow chimeras were infected with 2 × 10^6^ PFU LCMV Cl13 i.v. or left uninfected, and 24 h p.i., the spleens were analyzed for the frequency of cDCs (CD11b^+^ DCs) (B), the frequency of pDCs (C), and CD86 expression by cDCs and pDCs (D). (E) Frequency and IFN-γ, Tbet, and granzyme B expression of NK cells. (F and G) Flt3L-derived BM DCs were stimulated with loxoribine in the presence or absence of 50 ng/ml rIL-27. The data represent 4 mice per condition from 2 independent experiments (A); 6 uninfected controls, 5 uninfected *Il27ra*^−/−^ chimeras, 4 infected controls, and 8 infected *Il27ra*^−/−^ chimeras from 2 independent experiments (B to E); and 3 cultures per condition representative of the results of 3 independent experiments (F and G). The error bars indicate SEM. **, *P* < 0.01; *, *P* < 0.05.

To dissect IL-27-mediated restriction of viral replication that subsequently impacts innate cell numbers and function versus direct regulation of innate cells by IL-27, we first investigated a putative role of IL-27 in directly controlling LCMV growth independently of innate immune cells. Pretreatment of LCMV-susceptible cells (i.e., A549 cells) with increasing doses of recombinant IL-27 (rIL-27) before LCMV Cl13 infection had no effect on viral replication (Fig. 5A). This suggested that IL-27 could not increase an antiviral state in LCMV-susceptible cells and that the enhanced viral growth early after chronic LCMV infection was unlikely to have been a consequence of cell-intrinsic defective virus control in infected cells and may instead have resulted from the above-mentioned altered innate cell responses.

To further discern cell-intrinsic versus cell-extrinsic innate cell defects in the absence of il27ra signaling, we generated CD45.1:*Il27ra*^−/−^ and CD45.1:C57BL/6 WT mixed bone marrow chimeras and analyzed DCs and NK cells 24 h after LCMV Cl13 infection. *Il27ra*^−/−^ cDCs were slightly reduced in frequency compared to both cDCs from the CD45.1^+^ compartment and CD45.2^+^ DCs from control chimeras (Fig. 5B), but this was not observed in nonchimeric *Il27ra*^−/−^ mice (Fig. 3E). In contrast, the more pronounced intrinsic defect in the numbers of *Il27ra*^−/−^ pDCs 24 h after LCMV Cl13 infection than in WT pDCs (Fig. 5C) was in agreement with the reduced pDC accumulation observed in the *Il27ra*^−/−^ mice 1 day p.i. (Fig. 3E). CD86 expression by both pDCs and cDCs, which we had seen decreased in nonchimeric WSX-1-deficient mice (Fig. 3G), was actually significantly increased in *Il27ra*^−/−^ mice compared to their CD45.1^+^ WT counterparts in bone marrow chimeras, but this effect was also observed in the CD45.1:WT control chimeras (Fig. 5D). Intrinsic IL-27R signaling appeared to have no role in controlling NK cell frequency or function, as measured by expression of Tbet, IFN-γ, and granzyme B at day 1 after LCMV Cl13 infection (Fig. 5E). None of the above-mentioned DC or NK cell phenotypes appeared to be affected in *Il27ra*^−/−^ versus WT cells before infection (Fig. 5B to E).

Given the above-mentioned cell-intrinsic effect of IL-27 in pDCs, we next investigated the direct effect of recombinant IL-27 on uninfected BM-Flt3L-derived DCs (which include pDCs) in the absence or in the presence of the TLR7 ligand loxoribine, independently of the infectious environment. Under these conditions, we could not detect any effect of recombinant IL-27 on either unstimulated or TLR-7-stimulated pDC viability or numbers (Fig. 5F and data not shown), suggesting that the cell-intrinsic IL-27 induction of pDC maintenance observed *in vivo* may result from modulation of regulatory mechanisms that are not recapitulated in BM-derived pDCs. Furthermore, addition of rIL-27 to BM-derived DCs stimulated with loxoribine did not significantly affect IFN-I production, with only a slight increase reaching statistical significance compared to controls in 1 out of 3 independent experiments (Fig. 5G). Note that upon loxoribine stimulation, only BM-derived pDCs, but not BM-derived cDCs, produced IFN-I (data not shown).

Overall, these data showed that the majority of early defects seen in the innate immune responses in the global absence of WSX-1 after LCMV Cl13 were cell extrinsic. The exception was the effect on pDCs, which required IL-27 signaling to optimally maintain their numbers early after infection.

### *Il27ra* shapes the adaptive immune response to chronic LCMV.

Containment of persistent LCMV variants requires a combination of innate, CD8^+^, and CD4^+^ T cells and antibody-mediated immunity, as impairment of any one of these compartments can allow virus to persist indefinitely (3–5, 49). Given that early innate immune responses are crucial to prime and sustain the adaptive response to infection and the prior observation that late control of LCMV Cl13 persistence is WSX-1 dependent (43), we next sought to determine the effect of *Il27ra* deletion on the generation of LCMV-specific T cell and B cell responses.

**FIG 6:**
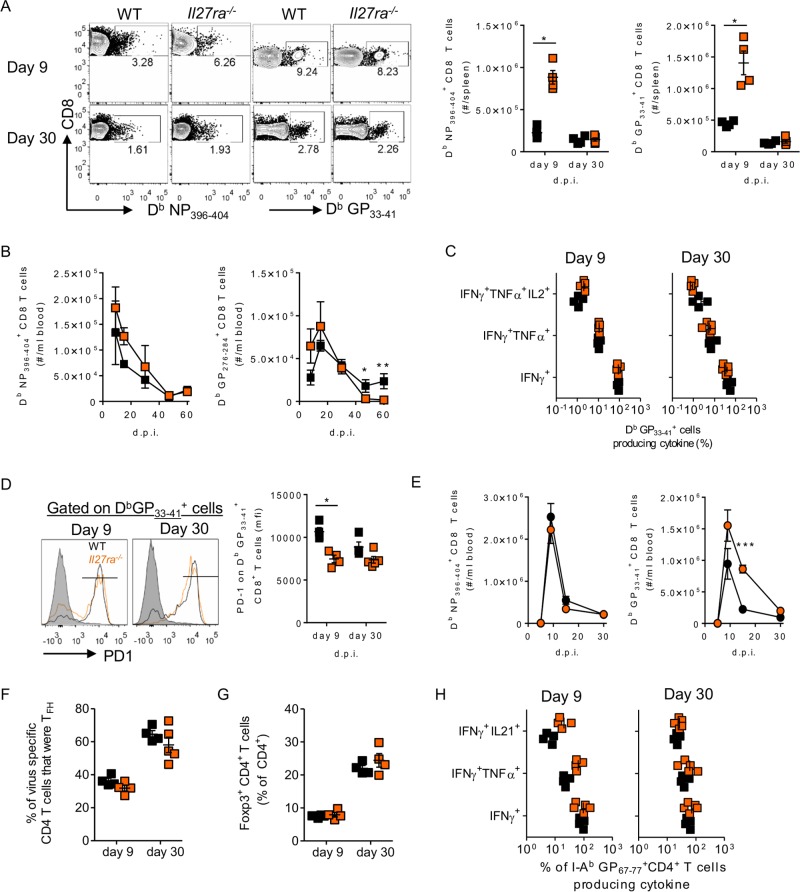
IL-27 signaling has limited effects on T cell responses during LCMV infection. WT and *Il27ra*^−/−^ mice were used throughout. (A to D) The numbers of splenic D^b^NP_396–404_^+^ and D^b^GP_33–41_^+^ CD8^+^ T cells (A), the numbers of circulating D^b^NP_396–404_^+^ and D^b^GP_276–284_^+^ CD8^+^ T cells (B), cytokine secretion by splenic CD8^+^ T cells after *ex vivo* GP_33–41_ peptide stimulation (C), and PD-1 expression by splenic D^b^GP_33–41_^+^ CD8^+^ T cells after LCMV Cl13 infection (D) were determined by flow cytometry. (E) Frequencies of D^b^NP_396–404_^+^ and D^b^GP_33–41_^+^ CD8^+^ T cells in the blood determined after infection with 2 × 10^6^ PFU of LCMV ARM i.v. (F to H) Frequencies of T_FH_s, gated as CXCR5^+^ ICOS^+^ SLAM^−^ CD4^+^ cells (F), Foxp3^+^ CD4 T cells (G), and cytokine-secreting CD4 T cells (H), in response to GP_67–77_ peptide stimulation determined in the spleens of LCMV Cl13-infected mice. The data represent ≥4 mice per group and are representative of the results of at least 2 independent repeats. The error bars indicate SEM. ***, *P* < 0.001; **, *P* < 0.01; *, *P* < 0.05.

IL-27 has been implicated in the proliferation and differentiation of antigen-specific CD8^+^ T cell responses (28, 50, 51). *Il27ra*^−/−^ mice had significantly fewer D^b^GP_276–284_^+^ CD8^+^ T cells than WT mice. In the spleen, there were significantly increased numbers of D^b^NP_396–404_^+^ and D^b^GP_33–41_^+^ CD8^+^ T cells at day 9 p.i. in the absence of WSX-1 (Fig. 6A). By day 30 p.i., however, the numbers of LCMV-specific CD8 T cells were similar in WT and knockout (KO) mice. *Il27ra*^−/−^ mice also had slightly increased numbers of blood LCMV-specific D^b^NP_396–404_^+^ and D^b^GP_276–284_^+^ CD8^+^ T cells at days 9 and 15 p.i., but these differences did not reach statistical significance (Fig. 6B). The differences declined as the infection progressed, and by day 45 p.i., *Il27ra*^−/−^ mice had fewer circulating GP_276–284_-specific CD8 T cells than WT mice. The functional capacities of GP_33–41_-stimulated CD8^+^ T cells to hierarchically produce IFN-γ, TNF, and IL-2 were similar in WT and *Il27ra*^−/−^ mice at both days 9 and 30 p.i. (Fig. 6C). Despite this, PD-1 expression, a key marker of T cell exhaustion during chronic infection, by virus-specific CD8^+^ T cells was significantly lower in the absence of WSX-1 at day 9 p.i. and trended the same way at day 30 p.i. (Fig. 6D). In comparison to chronic LCMV infection, during acute LCMV infection, circulating D^b^NP_396–404_^+^ CD8^+^ T cell numbers were similar in WT and *Il27ra*^−/−^ mice throughout infection; however, D^b^GP_33–41_^+^ numbers were elevated in the absence of WSX-1 (Fig. 6E).

Gp130 on virus-specific CD4^+^ T cells is required for their survival and differentiation into T_FH_s during LCMV ARM or Cl13 infection under competitive conditions (43, 52). In *Il27ra*^−/−^ mice, however, the number of virus-specific CD4^+^ T cells was actually heightened at day 9 after LCMV Cl13 infection, though it returned to numbers similar to those of WT mice at day 30 p.i. (43). One remaining question from these studies was whether IL-27, given its importance in determining CD4^+^ T cell differentiation in other inflammatory and infectious environments (32, 34), regulated CD4^+^ T cell fate during LCMV infection. Neither T_FH_ nor Treg frequencies were affected by WSX-1 deletion at days 9 and 30 after LCMV Cl13 infection (Fig. 6F and G). At the same times, secretion levels of IFN-γ, TNF, and IL-21, a key antiviral cytokine and potential downstream target of IL-27, by virus-specific CD4^+^ T cells were all similar (Fig. 6H).

**FIG 7:**
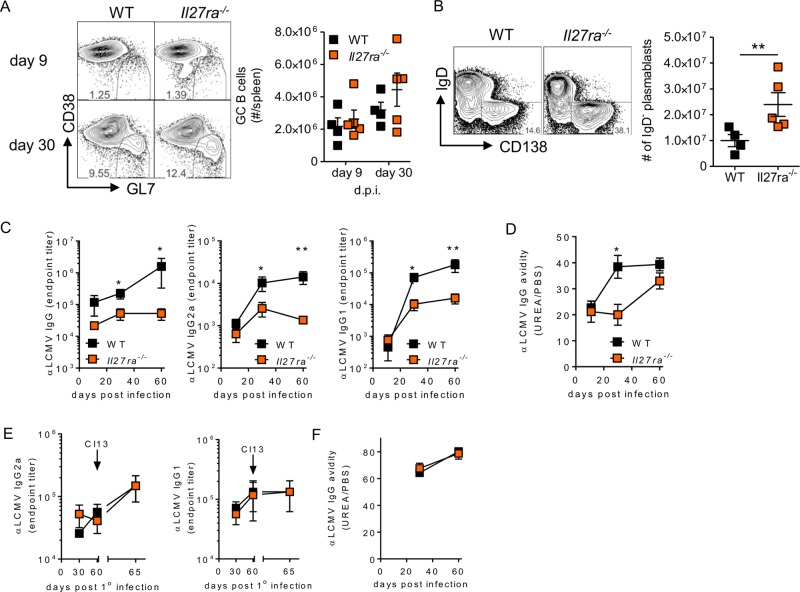
IL-27 deficiency results in reduced antibody responses in chronic, but not acute, infection. (A to D) WT and *Il27ra*^−/−^ mice were infected with 2 × 10^6^ PFU of LCMV Cl13 i.v., and the number of germinal center B cells (A); the number of CD138^+^ plasmablasts at day 9 p.i. (B); LCMV-specific IgG, IgG2a/c, and IgG1 (C); and IgG avidity (D) were measured in the sera. (E) WT and *Il27ra*^−/−^ mice were infected with 2 × 10^6^ PFU of LCMV ARM i.v., and levels of serum LCMV-specific IgG2a and IgG1 were determined before and after rechallenge with LCMV Cl13. (F) Avidity of LCMV-specific IgG in serum. The data are representative of the results of 2 independent experiments on ≥4 mice per time point per group. The error bars indicate SEM. **, *P* < 0.01; *, *P* < 0.05.

Virus-specific antibody responses are also vital for containment of chronic LCMV infection (5). Germinal center (GC) B cell numbers were similar in WT and WSX-1-deficient mice at days 9 and 30 after LCMV Cl13 infection (Fig. 7A). However, *Il27ra*^−/−^ mice did have a population of B cells that were CD38^−^ GL7^−^ in their spleens at day 9 p.i. that was not observed in WT mice. Further evaluation of B cell subsets revealed that the cells represented a subset of CD138^+^ B cells and that *Il27ra*^−/−^ mice had significantly more of these IgD^−^ plasmablasts than WT mice at day 9 postinfection (Fig. 7B). Perhaps counterintuitively, given the larger number of plasmablasts, *Il27ra*^−/−^ mice had slightly reduced LCMV-specific IgG by day 30 p.i., and absence of IL-27R led to deficiencies in both LCMV-specific IgG2a and IgG1 (Fig. 7C) and slightly affected IgG avidity at day 30 p.i., although it was similar to that seen in WT mice by day 60 (Fig. 7D). Loss of IL-27R signaling did not affect the amount, type, or quality of antibodies produced in response to acute LCMV ARM infection, nor was antibody induction on rechallenge with LCMV Cl13 affected (Fig. 7E and F).

**FIG 8:**
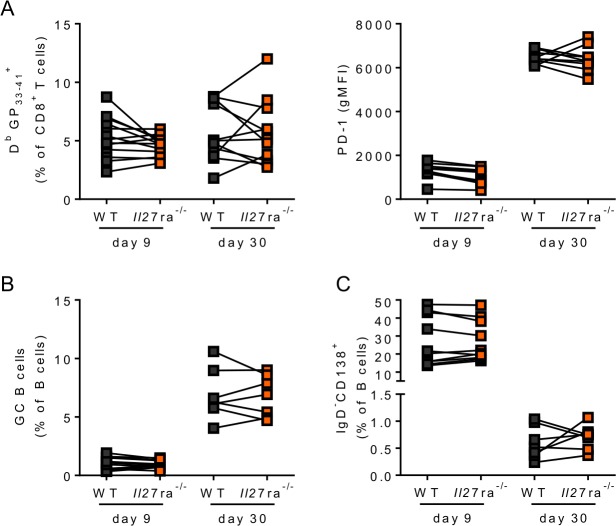
Intrinsic IL-27 signaling is not required for CD8 T cell or B cell differentiation. WT:*Il27ra*^−/−^ mixed bone marrow chimeras were infected with 2 × 10^6^ PFU LCMV Cl13 i.v., and at days 9 and 30 p.i., the frequencies of D^b^GP_33–41_^+^ CD8^+^ T cells and their PD-1 expression (A), GL7^+^ CD38^−^ germinal center B cells (B), and IgD^−^CD138^+^ plasmablasts (C) in the WT (CD45.1^+^) and *Il27ra*^−/−^ (CD45.2^+^) spleen compartments were determined. The data represent ≥7 mice from 3 independent experiments.

To determine if the changes in the adaptive immune response in the absence of *Il27ra* were a result of the altered viral and inflammatory environment or of direct signaling on B and T cells, we next analyzed responses in mixed bone marrow chimeras. In contrast to the cell-intrinsic IL-27R regulation of LCMV-specific CD4 T cell numbers late after LCMV Cl13 infection (43), we observed that there were similar frequencies of both virus-specific CD8^+^ T cells and their PD-1 expression in the WT and *Il27ra*^−/−^ compartments of CD45.1:*Il27ra*^−/−^ mixed BM chimeras at both days 9 and 30 after LCMV Cl13 infection (Fig. 8A). We also saw no significant difference in GC B cell or plasmablast frequencies at either time point p.i. (Fig. 8B and C).

Together, these data showed that IL-27 signaling affected adaptive responses by decreasing virus-specific antibody responses despite increasing the numbers of virus-specific CD8 T cells and plasmablasts. Notably, only from day 30 p.i. onward, when viral loads were once again significantly different in WT and *Il27ra*^−/−^ mice (43), did virus-specific antibodies begin to become adversely affected. Importantly, similar defects were not seen in the generation of immunity against LCMV ARM, and when wild-type and *Il27ra*^−/−^ GC B cells and plasmablasts were in the same environment, their frequencies were comparable. Although there may be some IL-27 intrinsic effects on parameters not studied here, our data suggested that the environment induced by IL-27R signaling is likely a key driver of antibody induction during chronic LCMV infection. Even if B cell extrinsic, however, the above-mentioned defective antibody responses could contribute to the inability of *Il27ra*^−/−^ mice to clear LCMV Cl13 infection.

## DISCUSSION

Containment of continuously replicating chronic viral infections requires a combined effort of innate cells, T cells, and antibodies (1). Here, we found that during chronic LCMV infection, IL-27 deficiency was associated with dysregulation of the innate immune response and failure to contain early viral replication. IL-27 was enhanced rapidly by DCs and other myeloid cells upon infection, with pDCs in particular upregulating IL-27 transcripts in a TLR7-dependent fashion. Early IL-27 signaling aided in promoting peak IFN-I elevation, the NK cell cytotoxic program, and DC activation. This aberrant inflammatory response was associated with disrupted adaptive immune responses at later stages of infection and lifelong viremia (43).

IL-27 signaling has a well-described role in T cell responses, where it regulates both the differentiation and function of CD4^+^ and CD8^+^ T cells, in particular promoting Tbet and IFN-γ production (25, 26, 28). IL-27 can also play a critical immunoregulatory role, suppressing IL-2 production by CD4 T cells (53) and promoting their IL-10 production (35–37). In accordance with this, IL-27R signaling has a negative impact on immune protection after chronic infections, such as Mycobacterium tuberculosis or murine cytomegalovirus (54, 55). In contrast, we have previously shown that IL-27R signaling is critical for promoting immune protection during chronic LCMV infection, where cell-intrinsic IL-27R signaling promotes the survival of virus-specific CD4^+^ T cells when IL-27R-deficient and -sufficient CD4^+^ T cells are placed in competition (43). Notably, ubiquitous IL-27R signaling deficiency resulted in increased numbers of virus-specific CD4^+^ T cells at day 9 p.i. (43), but here, we show that it did not affect their cytokine secretion, T_FH_ differentiation, or the frequency of Foxp3^+^ regulatory T cells, which IL-27 can modulate under different conditions (34, 43). Global loss of IL-27R signaling also increases the number of LCMV-specific CD8^+^ T cells after both chronic and acute infection, but as with the increase in CD4 T cell numbers, this is not a cell-intrinsic effect, and loss of IL-27R does not adversely affect the functional capacity of the CD8 T cell response. Although further studies may reveal additional IL-27 effects on T cells, our work highlights selected cell-intrinsic versus -extrinsic, as well as context-specific, roles of IL-27R signaling on T cell responses to a systemic persistent pathogen.

While we did not observe major alterations in T cell responses from WSX1-deficient mice by day 30 p.i., prior to the establishment of lifelong viral infection, there were more notable reductions in the quantity and quality of LCMV-specific antibodies. We and others have previously reported that antibody responses are critical for the control of chronic LCMV infection (5, 17, 56). IL-27 has been shown to influence T_FH_ formation and function in certain circumstances (34, 43), and it can also act directly on B cells. In human B cell cultures, IL-27 signaling has the capacity to regulate the proliferation of naive B cells, their differentiation into plasma cells, and isotype switch (57, 58), while in similar cultures using murine B cells, it was found to upregulate Tbet and promote IgG2a switch (59). In chronic LCMV infection, IL-27 signaling does not appear to diminish GC B cell differentiation, although it does limit the emergence of plasmablasts. Importantly, however, during LCMV Cl13 infection, IL-27 limited the production of LCMV-specific IgG2a and IgG1, along with overall IgG avidity. IL-27 signaling did not affect either the production or type of antibodies after acute LCMV infection, where viral clearance is maintained in the absence of IL-27, nor does cell-intrinsic IL-27 signaling appear to be important for GC B cell or plasmablast numbers. Taken together, our data show that intrinsic IL-27R signaling has modest effects on the outcome of multiple aspects of adaptive immune responses studied here. It is therefore likely that the reduced levels of LCMV-specific IgGs seen in chronically infected *Il27ra*^−/−^ mice resulted, at least in part, from their increased viral loads and inflammatory state.

Crucially, there were significant effects on the innate inflammatory response in the absence of IL-27 signaling. Notably, IFN-I production, which peaks 24 h after LCMV infection (21), was significantly reduced in the absence of IL-27 signaling. IFN-I promotes an antiviral state in cells through the induction of hundreds of interferon-stimulated genes (ISGs) via phosphorylation of STAT-1 and STAT-2 (46). A reduction in this process would explain the reduced virus control seen at early time points after LCMV Cl13 infection, and it is possible that high viral loads at early time points contribute to viral persistence at later stages. Signaling through IFN-αβR is critical for control of LCMV Cl13 persistence, and its absence results in enhanced depletion and exhaustion of virus-specific T cells (49), though sustained IFN-I signaling also has a deleterious effect on T cell responses (18, 19). Thus, it is conceivable that reduced IFN-I levels in *Il27ra*^−/−^ mice early after LCMV Cl13 infection could contribute to failure of virus control at later points postinfection. In this study, we did not evaluate whether IL-27R signaling influences chronic IFN-I signaling, as well. An alternative option is that IL-27 itself may drive a cell-intrinsic antiviral state that protects mice from LCMV infection. IL-27 can phosphorylate STAT-1 (25, 42) and has been shown to have antiviral activity against other persistent viral infections, including both HIV and hepatitis C virus (41, 42). During HIV infection, this process is related to IL-27's ability to upregulate a subset of ISGs independently of IFN-I (41) and to promote a number of anti-HIV molecules (60, 61); however, we show here that, at least when used *in vitro*, IL-27 appears to have no restrictive effect in LCMV growth, making this hypothesis less likely. Interestingly, however, IFN-I itself is also capable of driving IL-27-induced antiviral activity (62). During LCMV infection, IL-27 is produced by pDCs, cDCs, and other myeloid cells, the same cells reported to produce the majority of IFN-I (47, 48), and indeed, pDCs require TLR7 to produce both IFN-I (22) and IL-27. Furthermore, CD11b^+^ DCs and other CD11b^+^ myeloid cells from *Il27ra*^−/−^ mice had reduced *Ifna* and *Ifnb* transcription, while intrinsic WSX-1 signaling after LCMV Cl13 infection was also critical for maintenance of pDCs, which are major sources of IFN-I *in vivo* (22). Taken together, these findings suggest that an IL-27 positive-feedback loop may contribute to maintenance of pDC numbers very early after infection and to IFN-I production from myeloid cells and that disabling such a loop could compromise peak IFN-I responses and virus control.

IL-27 deficiency also affected NK cell and DC activities. The consequences of compromised DC maturation in the absence of IL27R signaling early after chronic LCMV infection are difficult to dissect, given that IL-27R-deficient mice also had heightened antigen burdens at these early time points. On the other hand, IL-27 can enhance NK cell antitumor activity, including upregulation of Tbet and granzyme B *in vivo* (38), both of which we found to require IL-27 for optimal upregulation after LCMV Cl13 infection. One surprising observation was that, in the absence of global IL-27R signaling, IFN-γ production by NK cells increased, but Tbet expression decreased. The IFN-γ gene is a target of Tbet in NK cells, and IFN-γ and Tbet are commonly induced by the same stimuli, although Tbet is not an absolute requirement for IFN-γ production by NK cells (63). In human NK cells, IL-27 can directly upregulate Tbet in NK cells, as it does in T cells (64); however, *in vivo*, there is a suggestion that IL-27 acts indirectly to promote NK cell activity (65). One hypothesis is that Eomes, another key regulator of NK cell function (66), compensates for the slightly lower expression of Tbet in the absence of IL-27R. Critically, however, *Il27ra*^−/−^ NK cells displayed none of the defects found in IL-27R-deficient animals when they were placed in the same environment as WT NK cells, showing that none of the defects observed in NK cells are the result of direct IL-27 signaling. One mechanism by which NK cell activity could be modulated is through promotion of IL-12 production by DCs; however, we found no difference in the levels of *Il12p35* in the spleen. However, we did find higher expression of *Il18* in *Il27ra*^−/−^ mice, and IFN-γ production by NK cells is known to be heavily induced by IL-18, synergistically with IL-12 (67, 68), which may explain the heightened IFN-γ production by NK cells from IL-27R-deficient infected mice. NK cell activity is traditionally considered to be antiviral (reviewed in reference 69). During chronic infection with LCMV, however, the dominant role of NK cells appears to be in curbing virus-specific T cell responses and preventing the development of immunopathology, rather than reducing the viral load (70, 71). Thus, the reduced NK cell cytotoxic program may also help explain the increased numbers of virus-specific CD8^+^ and CD4^+^ T cells that we observed in IL-27R-deficient mice.

In conclusion, our study demonstrated that ubiquitous IL-27R deficiency affected antiviral immunity at multiple junctures during a systemic chronic viral infection *in vivo*. In contrast to previous studies that focused on IL-27's ability to regulate T cells, we report here that IL-27R signaling largely influenced innate immunity and altered the inflammatory environment after infection, likely influencing long-term immunity. This was associated with IL-27's ability to promote very early, as well as late, pathogen containment during chronic viral infection.

## MATERIALS AND METHODS

### Mice and viral stocks.

WT C57BL/6, WT CD45.1^+^ (B6.SJL-*Ptprc^a^ Pepc^b^*/BoyJ), and *Il27ra*^−/−^ (WSX-1-deficient) mice were purchased from the Jackson Laboratory (Bar Harbor, ME). *Tlr7*^−/−^ mice were kindly provided by Shizuo Akira (Osaka University, Osaka, Japan). For mixed bone marrow chimeras, recipient mice received 1,000 rads and were reconstituted with 5.0 × 10^6^ (at a 50:50 ratio between the two donors) bone marrow cells intravenously (i.v.) The chimeras were then allowed to reconstitute for 8 weeks prior to infection. Mice were bred and maintained in a closed breeding facility, and mouse handling conformed to the requirements of the National Institutes of Health and the Institutional Animal Care and Use Guidelines of the University of California San Diego (UCSD). Unless otherwise stated, 6- to 8-week-old mice were infected i.v. with 2 × 10^6^ PFU of LCMV ARM or Cl13. Viruses were grown, identified, and quantified as described previously (2, 72).

### Purification of DCs.

Spleens were incubated with 1 mg/ml collagenase D for 20 min at 37°C and passed through a 100-μm strainer to achieve a single-cell suspension. Splenocytes were then incubated for 30 min with rat monoclonal antibodies (MAbs) specific for Thy1.2 and CD19, followed by magnetic cell sorting using anti-rat immunoglobulin-coated magnetic beads (Qiamag beads; Qiagen, Redwood City, CA) according to the manufacturer's instructions. The bead-negative fraction of cells were then stained with a panel of fluorescently conjugated MAbs, along with propidium iodide (PI), and fluorescence-activated cell sorting (FACS) purified using a BD ARIA II (BD) for pDCs (PI^−^ CD11c^intermediate/dim^ CD11b^−^ B220^+^ PDCA^+^), CD11b^+^ cDCs (PI^−^ CD11c^+^ B220^−^ CD11b^+^ CD8^−^), CD8^+^ DCs (PI^−^ CD11c^+^ B220^−^ CD11b^−^ CD8^+^), and other myeloid cells (PI^−^ CD11b^+^ CD11c^−^) after B (CD19), T (Thy1.2), and NK (Nk1.1) cell exclusion. Purities were >92%.

### Flow cytometry.

Flow cytometry was carried out as described previously (43). All antibodies and tetramers used for T cell identification and functional characterization were also identical to those used previously (43). Tetramers or monomers were kindly provided by the NIH Tetramer Core Facility (Atlanta, GA). For DCs and macrophages, the following anti-mouse antibodies (from eBioscience unless otherwise stated) were used: anti-Thy 1.2-peridinin chlorophyll protein (PerCP)-Cy5.5 (145-2C11), CD19-PerCP-Cy5.5 (eBio1D3), NK1.1-PerCP-Cy5.5 (PK136), CD11b-phycoerythrin (PE)-Cy7 (M1/70), B220-allophycocyanin (APC)-eF780 (RA3-6B2), PDCA-1-fluorescein isothiocyanate (FITC) (eBio927), CD86-PE (IT2.2; Biolegend), and CD8-eF450 (53-6.7). For NK cell identification, freshly isolated splenocytes were surface stained with anti-NK1.1-PerCP-Cy5.5 (PK136) and CD3e-eFluor450 (17A2). For Tbet analysis, the cells were then stained intranuclearly with anti-mouse/human-Tbet-FITC (4B10; Santa Cruz Biotech, Dallas, TX) after fixation with the Foxp3 permeabilization and fixation kit (eBioscience). For granzyme B, Lamp-1, and IFN-γ staining, splenocytes were incubated for 5 h at 37°C in the presence of 1 μg/ml brefeldin A and anti-mouse CD107a/b-FITC (1D4B, Invitrogen). NK cells were stained and fixed with 1% paraformaldehyde and permeabilized with saponin. Subsequently, they were stained with IFN-γ–APC (XMG1.2) and anti-human granzyme-B–PE (GB12; Invitrogen). Cells were acquired using the Digital LSR II flow cytometer (Becton Dickinson, San Jose, CA). Flow cytometric data were analyzed with FlowJo software (TreeStar; CA).

### Bone marrow-derived DCs.

BM cells were isolated from femurs and tibias, and a single-cell suspension was prepared and cultured for 7 to 8 days in the presence of 100 ng/ml Flt3L (Amgen, Thousand Oaks, CA, and Cell Dex Therapeutics, Needham, MA), as previously described (73). The cells were stimulated with 0.1 μM CpG B 1668 (Integrated DNA Technologies, San Diego, CA), 100 ng/ml LPS, 25 μg/ml poly(I · C), and 100 μM loxoribine (Invivogen).

### Real-time RT-PCR.

Total RNA from sorted cells was extracted using RNeasy Micro kits (Qiagen) and reverse transcribed into cDNA using Superscript III reverse transcriptase (RT) (Invitrogen). Total RNA from spleen homogenates was extracted with RNeasy minikits (Qiagen), and 1 μg of RNA was converted to cDNA using a Moloney murine leukemia virus (MMLV) reverse transcriptase kit (Promega, Madison, WI). cDNA quantification was performed with a real-time PCR detection system (Applied Biosystems, Carlsbad, CA) using SYBR green PCR kits. All the genes were normalized to *Gapdh* as previously described (21). For SYBR green, the following primers were used at 280 nM: *Ifnα 4*/*6/7* F, 5′-TATGTCCTCACAGCCAGCAG-3′, and R 5′-TTCTGCAATGACCTCCATCA-3′; *Ifnβ1* F, 5′-CTGGCTTCCATCATGAACAA-3′, and R, 5′-GAGGGCTGTGGTGGAGAA-3′; *Tnfα* F, 5′-CCCTCACACTCAGATCATCTTCT-3′, and R, 5′-GCTACGACGTGGGCTACAG-3′; *Il27p28* F, 5′-CTGTTGCTGCTACCCTTGCTT-3′, and R, 5′-CACTCCTGGCAATCGAGATTC-3′; *Ebi3* F, 5′-ACAACTGAGCCACACTGGGCA-3′, and R, 5′-CCACACCGAGCCTGTAAGTGG-3′; *LCMV GP* F, 5′-CATTCACCTGGACTTTGTCAGACTC-3′, and R, 5′-GCAACTGCTGTGTTCCCGAAA-3′; *LCMV NP* F, 5′-GCATTGTCTGGCTGTAGCTTA-3′, and R, 5′-CAATGACGTTGTACAAGCGC-3′; *Il18* F, 5′-GCCTCAAACCTTCCAAATCA-3′, and R, 5′-TGGATCCATTTCCTCAAAGG-3′; *Il12p35* F, 5′-AAATGAAGCTCTGCATCCTGC-3′, and R, 5′-TCACCCTGTTGATGGTCACG-3′; *Gapdh* F, 5′-TCCCACTCTTCCACCTTCGA-3′, and R, 5′-AGTTGGGATAGGGCCTCTCTT-3; human *GAPDH* F, 5′-TGATGACATCAAGAAGGTGGTGAAG-3′, and R, 5′-TCCTTGGAGGCCATGTGGGCCAT-3′. For *Il6*, TaqMan master mix (Applied Biosystems) was used in conjunction with the appropriate primers and probe from the Universal Probe Library (Roche).

### LCMV-specific antibody ELISAs.

LCMV-specific enzyme-linked immunosorbent assays (ELISAs) were done as we and others have previously described using antigen prepared by purifying LCMV on a renografin gradient (17). Endpoint titers were calculated by determining the lowest dilution at which a specific antibody was at least 2 times the standard deviation (SD) above background. For avidity assays, an LCMV-specific Ig ELISA was performed. After incubation of serial dilutions, sera on LCMV-coated plates were washed 3 times with 8 M urea or phosphate-buffered saline (PBS) prior to addition of a horseradish peroxidase (HRP)-conjugated anti-mouse Ig secondary antibody (Southern Biotech, Birmingham, AL). The percent binding was then determined as the optical density of urea (OD_urea_) divided by the OD_PBS_ times 100 at a 1/4,000 serum dilution when both were detectable above baseline.

### Cytokine detection.

Total IFN-I bioactivity was measured by luciferase bioassay with reference to a recombinant mouse IFN-β standard (Research Diagnostics, Concord, MA), using an L-929 cell line transfected with an interferon-sensitive luciferase, as previously described (74). IL-27, TNF-α, and IL-6 were measured by ELISA (eBioscience, La Jolla, CA).

### *In vitro* infection.

A549 cells (ATCC) were cultured in Dulbecco's modified Eagle's medium (DMEM) (Lonza, Walkersville, MD, USA) supplemented with 2 mM l-glutamine, 50 U ml^−1^ penicillin, and 50 mg ml^−1^ streptomycin (Gibco, Grand Island, NY, USA), plus 10% heat-inactivated fetal bovine serum (Lonza). The cells were maintained in 175-cm^2^ flasks at a density of 0.5 × 10^6^ to 1 × 10^6^ cells ml^−1^ in a total volume of 30 ml. A total of 1 × 10^6^ A549 cells were left untreated or pretreated for 1 h with 20 or 100 ng ml^−1^ human recombinant IL-27 (Biolegend). The cells were subsequently infected with LCMV Cl13 at a multiplicity of infection (MOI) of 0.05 or 0.5. After 1 day, the cells were washed with PBS and collected in lysis buffer (Qiagen) for RNA analysis.

### Statistics.

Statistical analysis was carried out using Graphpad Prism 5.0 (Graphpad, La Jolla, CA). For comparisons between 2 groups, a nonparametric Mann-Whitney U test was performed. For comparison of multiple groups, a Kruskal-Wallis test, followed by Dunn's comparison test, was used. For pairwise analysis, a Wilcoxon test was used.
